# Expression of the calcium-activated potassium channel in upper and lower segment human myometrium during pregnancy and parturition

**DOI:** 10.1186/1477-7827-7-27

**Published:** 2009-04-05

**Authors:** Lu Gao, Binghai Cong, Lanmei Zhang, Xin Ni

**Affiliations:** 1 Department of Physiology, Second Military Medical University, Shanghai 200433, PR China; 2 Department of Gynecology and Obstetric, Navy General Hospital, Beijing 100037, PR China

## Abstract

**Background:**

Large conductance calcium-activated potassium channel (BKCa) plays an important role in the control of uterine contractility during pregnancy. The change from uterine quiescence to enhanced contractile activity may be associated with the spatial and temporal expression of BKCa within myometrium. The objectives of this study were to examine the expression of BKCa alpha- and beta-subunit in upper segment (US) and lower segment (LS) regions of uterus, and to investigate for the possibly differential expression of these proteins in US and LS myometrium obtained from three functional states: (1) non-pregnant (NP); (2) term pregnant not in labour (TNL) and (3) term pregnant in labour (TL).

**Methods:**

Myometrial biopsies were collected from non-pregnant women at hysterectomy and pregnant women at either elective caesarean section or emergency caesarean section. Protein expression level and cellular localization of BKCa alpha- and beta-subunit in US and LS myometrium were determined by Western blot analysis and immunohistochemistry, respectively.

**Results:**

BK_Ca _alpha- and beta-subunit were predominantly localized to myometrial smooth muscle in both US and LS myometrium obtained from non-pregnant and pregnant patients. The level of BKCa alpha-subunit in US but not in LS was significantly higher in NP myometrium than those measured in myometrium obtained during pregnancy. Lower expression of BKCa alpha-subunit in both US and LS was found in TL than in TNL biopsies. Expression of beta-subunit in both US and LS myometrium was significantly reduced in TL group compared with those measured in TNL group. There was no significant difference in BKCa beta-subunit expression in either US or LS between NP and TNL group.

**Conclusion:**

Our results suggest that expression of BKCa alpha- and beta-subunit in pregnant myometrium is reduced during labour, which is consistent with the myometrial activity at the onset of parturition.

## Background

During most of pregnancy, myometrium activity is characterized by poorly coordinated contractures. In late pregnancy, the uterus undergoes preparedness for the stimuli that lead to contractility and labour [1,2]. The mechanisms that initiate labour in women, particularly the molecular processes that convert the myometrium form a state of relative quiescence to the activated and contractile state, are not well understood. An understanding of these processes, at the molecular and cellular level, is essential to developing novel therapeutic strategies for management of associated clinical problems such as preterm labour that accounts for 85% of all perinatal complications and death.

It has been known that uterine myometrial contractility at term is triggered by a number of physiological signals, which orchestrate changes in uterine excitability via ion-channel modulation [3,4]. Potassium channels are central to regulation of cell membrane potential and contractility of smooth muscle [4,5]. Among the diverse families of K^+ ^channels, the large conductance calcium-activated potassium channel (BK_Ca_) is the predominant K^+^-channel type expressed in human myometrium [6,7]. This channel is activated by membrane depolarization and also by an increase in the intracellular calcium concentration, thereby playing a pivotal role in the modulation of uterine contractility and myometrial calcium homeostasis [3,4,8-10]. Electrophysiological studies have demonstrated changes in BK_Ca _activity during pregnancy. It was reported by Wang et al. [11] that the contribution of BK_Ca _channels to the total outward K^+ ^currents was reduced by about 10% in pregnant myocyte near term compared with non-pregnant myocyte. Khan and colleagues had shown that the sensitivity of Ca^2+ ^and voltage of this channel in human pregnant myometrium was lost at the time of labor [7]. A number of studies suggested altered BK_Ca _expression in myometrium during pregnancy and parturition. Song et al. [12] worked on rat and found that BK_Ca _is decreased by 60% in the myometrium of pregnant rats at the end of pregnancy. Benkusky and co-workers [13] reported that BK_Ca _in mouse myometrium is increased during pregnancy and diminished in post-partum. Studies by Khan's group demonstrated BK_Ca _expression in lower segment of human term myometrium and found it is decreased in labour onset [14,15].

It has been implicated that there is a functional regionalization in the myometrium during pregnancy and labour. The upper segment (US) region of the uterus expands to accommodate the growing fetus and then at labour contract to cause expulsion of the fetus, while the lower segment (LS) may maintain a relative quiescence to allow passage of the fetus [16]. Current data about BK_Ca _expression in human myometrium during pregnancy are restricted to the LS [14,15,17]. There is no information concerning the expression of BK_Ca _in the different region of uterus during pregnancy and labour that would support its role in the regulation of uterus contractions.

The objectives of the present study were to determine the regional distribution of the BK_Ca _channel in human non-pregnant and term myometrium before and during parturition. Protein levels of BK_Ca _channel were also examined by Western blotting to establish whether the expression of these proteins is changed in pregnancy and labour.

## Methods

### Tissue Collection

Paired upper and lower uterine segmental myometrial tissues from pregnant and non-pregnant women were collected in Navy General Hospital, the teaching hospital of Second Military Medical University, Beijing, China. Approval of this study was granted by human ethic committee of Navy General Hospital as well as human ethic committee of Second Military Medical University. Written informed consent was obtained from each participant.

Non-pregnant myometrium tissues were obtained from premenopausal, normal, cycling women (mean age 41 ± 4.3 yr, n = 8) undergoing hysterectomy for fibroids. Pregnant myometrial biopsies were collected at cesarean section form the following groups of pregnant women (37–42 wk): term no labour (mean age, 25 ± 2.2 yr, mean gestational age,39 ± 1.7 weeks, n = 10) and term labour (mean age 26 ± 2.3 yr, mean gestational age, 39 ± 1.6 weeks n = 10). Labour was defined as regular contractions (<5 min apart) plus membrane rupture and cervical dilation (>3 cm) with no augmentation (oxytocin or PG administration). Indications for cesarean section included breech presentation, placenta previa, previous cesarean section, cephalopelvic disproportion, failure of labour to progress, fetal distress, or maternal request. None of the women included in this study had evidence of underlying disease (e.g. hypertension, diabetes, preeclampsia, intrauterine growth restriction, etc.). LS uterine samples were collected from the upper margin of the LS uterine incision, while US uterine samples were taken from just below fundus. Because there is no LS in non-pregnant uterus, tissues taken from the isthmus of non-pregnant uterus were treated to be equivalent to the LS of pregnant uterus. All non-pregnant tissues were taken from the normal part of uterus without fibrosis contamination. Collected samples were placed in phosphate-buffered saline on ice and transported to the laboratory. For Western blot analysis, tissues were then frozen immediately in liquid nitrogen and stored at -80°C. For immunohistochemical analysis, the biopsies were placed in 10% phosphate buffered formalin.

### Immunohistochemistry

Immunohistochemical investigations were performed with the Histostain-SP kit (Zymed, San Franscisco, CA), which uses a biotinylated second antibody, a horseradish peroxidase-streptavidin conjugate, and a substrate-chormogen mixture to demonstrate antigen in the tissue. The specific antibodies, anti-α_1184–1200 _and anti-β_1–191_, were purchased from Alomone Labs (Alomone Labs Ltd. Jerusalem, Israel) and Santa Cruz Biotechnology (Santa Cruz Biotechnology, Inc. Santa Cruz, CA), respectively. The anti-α_1184–1200 _antibody recognizes amino acid residues 1184–1200 at the C terminus of the α-subunit. Anti-β_1–191 _antibody recognizes full length residues of β-subunit. Paraffin sections (5 μm) were cut, rehydrated and microwaved in citric acid buffer to retrieve antigens. After inhibition of endogenous peroxidases with 3% H_2_O_2_, unspecific antibody binding was blocked with 10% rabbit serum for 30 min. The tissue sections were then incubated with specific antibody against BK_Ca _α- or β-subunit (1:500). The bound antibodies were detected with the biotin-streptavidin-peroxidase system (UltraSensitive-SP-kit, MaiXin Biotechnology, Fuzhou, China) using diaminobenzidine (Sigma) as chromogen. Counterstaining was performed with hemalum. Negative controls were performed by substituting primary antibody with a normal serum in same dilution. To confirm the specificity of primary antibody, preabsorption of the primary antibody with a ten-fold excess of the blocking peptides was performed.

### Western blot analysis

Approximately 70 mg of human myometrial tissue was homogenized in ice-cold lysis buffer consisting of 60 mM Tris-HCl, 2% sodium dodecyl sulfate (SDS), 10% sucrose, 2 mM phenylmethylsulfonyl fluoride (Merck, Darmstadt, Germany), 1 mM sodium orthovanadate (Sigma-Aldrich), 10 μg/ml aprotinin (Bayer, Leverkusen, Germany). Lysates were then quickly ultrasonicated in ice bath, boiled 5 min at 95 C, and stored at -80°C until used. Protein concentrations were measured using a modified Bradford assay and samples were diluted in sample buffer (250 mM Tris-HCl (pH 6.8), containing 4% SDS, 10% glycerol, 2% β-mercaptoethanol, and 0.002% bromophenol blue) and boiled for a further 5 min. Samples were separated on an SDS-8% polyacrylamide gel, and the proteins were electrophoretically transferred to a nitrocellulose filter at 300 mA for 1.5 h in a transfer buffer containing 20 mM Tris, 150 mM glycine, and 20% methanol. The filter was then blocked in TBS containing 0.1% Tween-20(TBST) and 5% dried milk powder (wt/vol) for 2 h at room temperature. After three washes with TBST, the nitrocellulose filters were incubated with primary antibody for BK_Ca _α- or β-subunit (1:500) at 4°C overnight. After another three washes with TBST, the filters were incubated with a secondary horseradish peroxidase-conjugated IgG (1:1000) for 1 h at room temperature and further washed for 30 min with TBST. Immunoreactive proteins were visualized using the enhanced chemiluminescence Western blotting detection system (Santa Cruz). The light-emitting bands were detected with X-ray film. The resulting band intensities were quantitated using an image scanning densitometer (Furi Technology, Shanghai, China). To control sampling errors, the ratio of band intensities to β-actin was obtained to quantify the relative protein expression level.

### Statistical analysis

Protein levels of BK_Caα _– and β-subunit were determined by densitometric analysis (Furi Technology, Shanghai, China). Peak count values were expressed as densitometric units. The data are presented as mean ± SEM. All data were tested for homogeneity of variance by Bartlett's test. The results indicated that the data were normally distributed. Individual comparisons were made by Student's t-test for paired data. One-way ANOVA with Student-Newman-Keuls was used for multiple comparisons. *P*-value of <0.05 was considered to be significant.

## Results

### Expression of the α- and β-subunit of BK_Ca _in non-pregnant and pregnant myometrium

**Figure 1:**
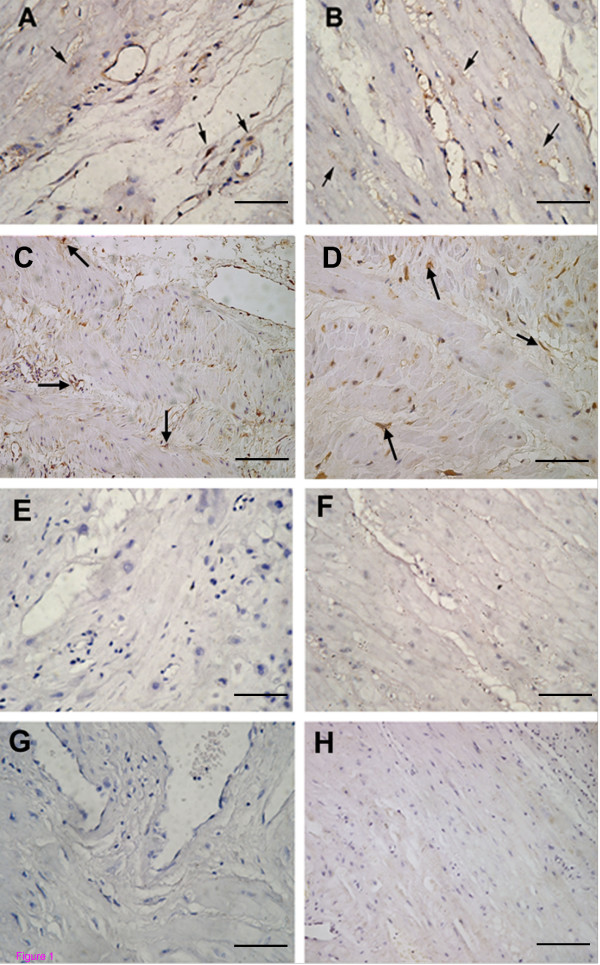
**Immunolocalization of BK_Ca _channel α- and β-subunit in non-pregnant myometrium**. Positive staining for the α-subunit of BK_Ca _(arrow) in (A) US myometrium and (B) LS myometrium. Positive staining for the β-subunit (arrow) in (C) US myometrium and (D) LS myometrium. (E-H) Negative controls. The primary antibody was substituted with (E) normal rabbit serum or (G) PBS. Sections were stained with (F) α-subunit preabsorption antibody or (H) β-subunit preabsorption antibody. Original magnification ×400.

**Figure 2:**
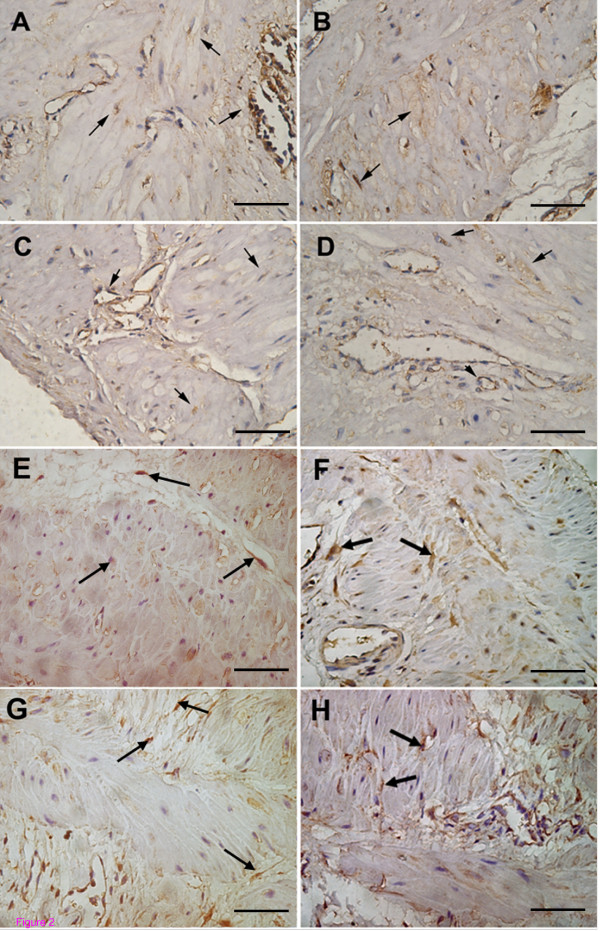
**Immunolocalization of BK_Ca _channel α- and β-subunit in myometrium from pregnant women at term in labour or not in labour**. A-D shows representative sections for positive staining for α-subunit in (A) US myometrium not in labour, (B) LS myometrium not in labour, (C) US myometrium in labour and (D) LS myometrium in labour. E-H shows representative sections for positive staining for β-subunit in (E) US myometrium not in labour, (F) LS myometrium not in labour, (G) US myometrium in labour and (H) LS myometrium in labour. Arrow: positive staining. Original magnification ×400.

Positive immunoreactivity for BK_Ca _α-and β-subunit was identified in both US and LS myometrium from non-pregnant and pregnant women. Immunohistochemistry revealed that BK_Ca _α- and β-subunit were predominantly localized to smooth muscle cells of myometrium. Smooth muscle cells lining blood vessel were also positively stained for these proteins (Fig 1 and 2). Immunoreactivity was abolished when the antibody was preabsorbed with excess peptide, thereby confirming the specificity of antibody (Fig 1E and 1F).

**Figure 3:**
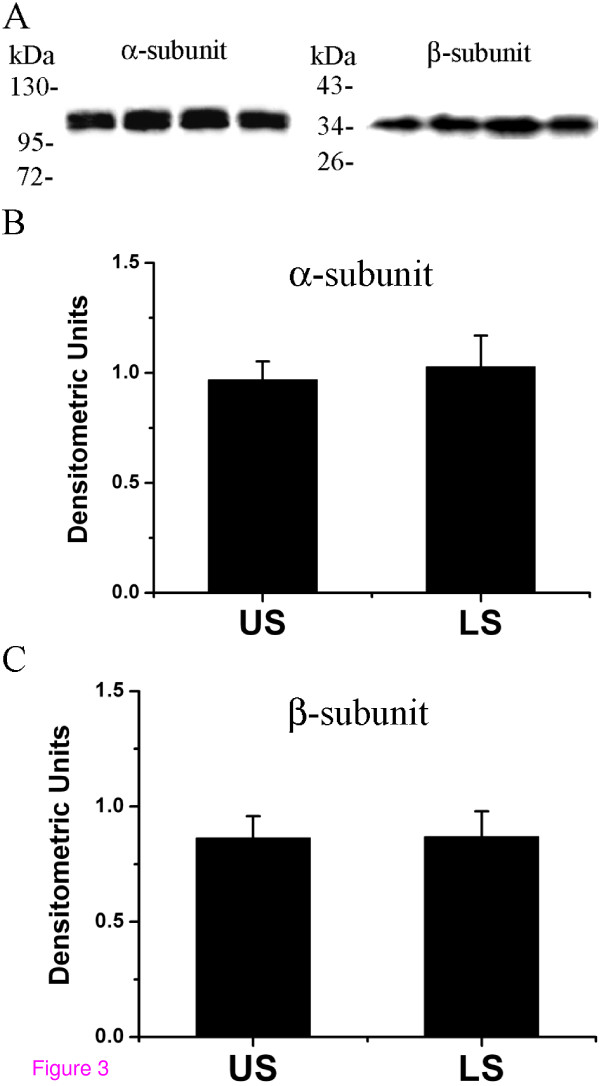
**Western blot analysis of BK_Ca _α- and β-subunit in pregnant US and LS myometrium**. (A) Representative immunoblots showing the expression of the 110 kDa α-subunit and the 35 kDa β-subunit in human myometrium. (B, C) The expression values of α- and β-subunit from all the pregnant patients was combined to given an overall expression profile in the US and LS. Data were expressed as mean ± SEM.

Western blot analysis, using α- and β-subunit-specific antibodies, detected bands of 110 and 35 kDa, respectively (Fig 3A). To given an overall expression profile in the US and LS, the expression values from all the pregnant patients were combined. When the overall expression level of each protein was compared in the pregnant upper and lower myometrium samples, there were no significant differences in either α- or β-subunit levels between US and LS myometrium (Fig 3B and 3C).

### Pregnancy and labour associated changes in the expression of BK_Ca _α-subunit

**Figure 4:**
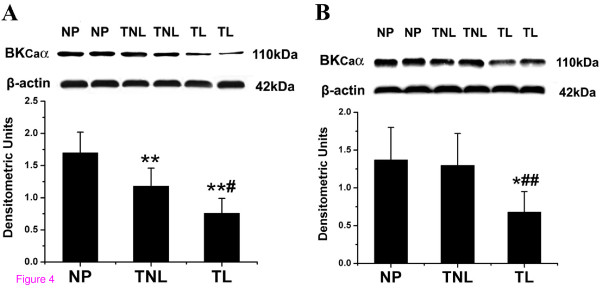
**Semiquantitation of Western blot signals of BK_Ca _α-subunit in US and LS myometrium**. Myometrial tissues were obtained from non-pregnant women (n = 8) and pregnant women at term before the onset of labour (n = 10) or during active labour (n = 10). (A) Levels of α-subunit in US samples. (B) α-subunit expression in LS samples. Representative protein bands were presented on the top of the histogram. Data were expressed as mean ± SEM. *P < 0.05, **P < 0.01 with NP; #*P *< 0.05, ##*P *< 0.01 with TNL.

Within US myometrium, the expression level of α-subunit protein was significantly down-regulated in pregnant samples compared to that of the non-pregnant samples (NP versus all other groups, *P *< 0.01, Fig 4A). It was further decreased in TL samples compared to TNL samples (*P *< 0.05, Fig. 4A).

Within LS, however, no significant differences in the expression of α-subunit protein were observed among NP and TNL groups. This protein was significantly down-regulated in TL samples compared to that in TNL samples (*P *< 0.01, Fig. 4B).

### Pregnancy and labour associated changes in the expression of BK_Ca _β-subunit

**Figure 5:**
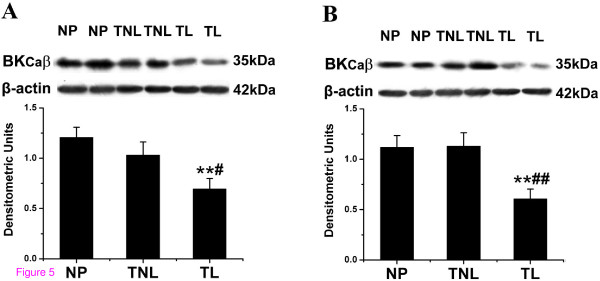
**Semiquantitation of Western blot signals of BK_Ca _β-subunit in US and LS myometrium**. Myometrial biopsies were obtained from nonpregnant women (n = 8) and pregnant women at term not in labour (n = 10) or during active labour (n = 10) (A) BK_Ca _β-subunit expression in US, (B) BK_Ca _β-subunit level in LS. Representative protein bands were presented on the top of the histogram. Data were expressed as mean ± SEM. **P < 0.01 with NP; #*P *< 0.05, ##*P *< 0.01 with TNL.

Within US, expression of β-subunit protein was significantly decreased in TL group (TL versus TNL, *P *< 0.05, TL versus NP, *P *< 0.01, Fig. 5A). Within LS, β-subunit protein level was significantly lower in TL group than that in TNL or NP group (*P *< 0.01, Fig. 5B). No marked changes in either US or LS were observed in pregnancy (NP versus TNL).

## Discussion

The present study demonstrated, for the first time, the expression of α- and β-subunit of BK_Ca _in paired US and LS tissues from non-pregnant and pregnant women. BK_Ca _α- and β-subunit were mainly localized to myometrial smooth muscle cells in the US and LS. The levels of these proteins were significantly down-regulated in both US and LS at the time of labour.

Studies by Khan's group showed that the expression of both BK_Ca _α- and β-subunit in LS region of uterus was significantly reduced during labour [14,15]. Curley et al. [17] also found decreased mRNA level of the α-subunit of BK_Ca _in LS with labour onset. In consistence with the above studies, the present study also showed the labour associated decrease in the expression of α- and β-subunit proteins within LS myometrium. In addition, we also investigated the localization and expression pattern of BK_Ca _within US during pregnancy and labour. Our data suggest that α- and β-subunit of BK_Ca _in US region is significantly down-regulated at the onset of labour.

Current data outlining pregnancy associated expression of BK_Ca _protein in LS region of human uterus are not fully consistent. Zhou et al. [18] reported that BK_Ca _α-subunit protein in LS region remained virtually unchanged in pregnant human myometrium, compared to non-pregnant myometrium, whereas β-subunit protein was lower in pregnant tissues than in non-pregnant tissues. Matharoo-Ball et al. [15] showed that BK_Ca _α-subunit was increased whereas β-subunit was decreased during pregnancy compared to the non-pregnant state. However, no studies to date appear to have examined BK_Ca _in US myometrium of pregnant and non-pregnant women. In the present study, within LS region, no significant pregnancy associated changes in the expression of either α- or β-subunit were observed. Within US, α-subunit expression was decreased in pregnancy. In animal studies, a decrease in BK_Ca _α-subunit protein levels close to or at term in rat myometrium [12] and an increase in the expression of BK_Ca _α and β1 subunit throughout gestation in mouse myometrium [19] have been reported.

A lot of studies indicate differential expression of a variety of proteins including connexin-43, G proteins and prostaglandin receptors between US and LS myometrium with labour [20-24], which support the idea that increased contractility of the fundus compared to the lower segment during labour. However, similar expression levels of a few contraction-associated proteins (CAPs) in US and LS with labour have also been reported [21,25,26]. For instance, Havelock et al. [26] showed that S100A9 mRNA was up-regulated in both US and LS during labour. Our results indicate that the similar expression pattern of BK_Ca _in US and LS is occurred with the onset of labour. Together, it suggests that the contractile activity of US or LS region is not likely to be determined by a single specific protein but rather by a combination of all CAPs.

The smooth muscle BK_Ca _channel is formed by tetrameric assembly of an α subunit and an accessory β-subunit [27]. The α-subunit forms the functional BK_Ca _channel [28,29]. The presence of the β-subunit confers BK_Ca _with higher Ca^2+ ^and voltage sensitivity [10,30]. Thus, reduced expression of BK_Ca _α- and β-subunit would permit an increase intracellular Ca^2+ ^levels without permit an increase opposing K^+ ^conductance, thereby enhancing contractility of smooth muscle. Because the BK_Ca _channel is particular abundant in uterine myocytes, a reduction of this channel in protein level would translate into a considerable shift toward electrical excitability in the human uterus and likely promote contractile activity.

## Conclusion

Our results indicate that BK_Ca _α- and β-subunit are predominately localized to myometrial cells in US and LS region of pregnant and non-pregnant human uterus. It has been implicated that, at the time of labour, the uterus differentiates into the highly contractile activity. Because BK_Ca _channel play a pivotal role in the modulation of uterine excitability and contractility, the down-regulation of BK_Ca _channel expression in myometrium following labour may contribute, in part, to the enhanced contractility during parturition.

## Competing interests

The authors declare that they have no competing interests.

## Authors' contributions

LG and BC carried out all experimental work. LZ recruited patients, organized the collection of tissues. XN conceived of the study, and participated in its design and coordination. All authors read and approved the final manuscript.
